# A comparison of the impact on neuronal transcriptome and cognition of rAAV5 transduction with three different doses in the mouse hippocampus

**DOI:** 10.3389/fnmol.2023.1195327

**Published:** 2023-07-14

**Authors:** Yi-Si Liu, Meng-Ling Wang, Neng-Yuan Hu, Zi-Ming Li, Jia-Li Wu, Hao Li, Jing-Ting Li, Xiao-Wen Li, Jian-Ming Yang, Tian-Ming Gao, Yi-Hua Chen

**Affiliations:** State Key Laboratory of Organ Failure Research, Key Laboratory of Mental Health of the Ministry of Education, Guangdong Province Key Laboratory of Psychiatric Disorders, Department of Neurobiology, Guangdong-Hong Kong-Macao Greater Bay Area Center for Brain Science and Brain-Inspired Intelligence, School of Basic Medical Sciences, Southern Medical University, Guangzhou, China

**Keywords:** rAAV, transcriptome, learning and memory, anxiety, hippocampus

## Abstract

**Introduction:**

Recombinant adeno-associated viruses (rAAVs) are widely used in genetic therapeutics. AAV5 has shown superior transduction efficiency, targeting neurons and glial cells in primate brains. Nonetheless, the comprehensive impact of AAV5 transduction on molecular and behavioral alterations remains unexplored. This study focuses on evaluating the effects of AAV5 transduction in the hippocampus, a critical region for memory formation and emotional processes.

**Methods:**

In this experiment, fluorescence-activated cell sorting (FACS) was utilized to isolate the mCherry-labeled pyramidal neurons in the hippocampus of CaMkIIα-cre mice following three different doses rAAV5-mCherry infusion after 3 weeks, which were then subjected to RNA sequencing (RNA-seq) to assess gene expression profiles. The cytokines concentration, mRNA expression, and glial response in hippocampi were confirmed by ELASA, digital droplet PCR and immunohistochemistry respectively. Locomotion and anxiety-like behaviors were elevated by Open Field Test and Elevated Plus Maze Test, while the Y-Maze were used to assessed spatial working memory. Recognition memory and fear responses were examined by the Novel Object Recognition Test and Fear Conditioning Test, respectively.

**Results:**

We found that 2.88 × 10^10^ v.g rAAV5 transduction significantly upregulated genes related to the immune response and apoptosis, and downregulated genes associated with mitochondrial function and synaptic plasticity in hippocampal pyramidal neurons, while did not induce neuronal loss and gliosis compared with 2.88 × 10^9^ v.g and 2.88 × 10^8^ v.g. Furthermore, the same doses impaired working memory and contextual fear memory, without effects on locomotion and anxiety-related behaviors.

**Discussion:**

Our findings highlight the detrimental impact of high-dose administration compared to median-dose or low-dose, resulting in increased neural vulnerability and impaired memory. Therefore, when considering the expression effectiveness of exogenous genes, it is crucial to also take potential side effects into account in clinical settings. However, the precise molecular mechanisms underlying these drawbacks of high-dose rAAV5-mCherry still require further investigation in future studies.

## Introduction

In preclinical and clinical studies involving *in vivo* genetic therapeutics and genetic modification, recombinant adeno-associated viruses (rAAVs) are currently the most commonly employed vehicles. During neurosurgical procedures involving the delivery of viral vectors to the brain, the objective is to minimize perturbation of the brain tissue while achieving efficient gene delivery for the desired duration. Using viral vectors with high tropism for the target cell type and wide transduction area can enable the delivery of the lowest possible vector dose, which reduces tissue damage, undesired spread, and the risk of genomic integration.

The adeno-associated virus 2 (AAV2) was the first AAV to be cloned at the molecular level and transformed into a replication-deficient vector, and it has been widely studied for its potential in gene transfer applications. Clinical trials for gene therapy aimed at treating brain diseases, such as Alzheimer's disease, Canavan's disease, and Parkinson's disease, are either underway or in progress, utilizing the well-researched rAAV2 (Hudry and Vandenberghe, 2019). However, its modest transduction levels and insufficient non-neuronal transduction have also been perceived as limitations (Burger et al., 2004; Taymans et al., 2007; Aschauer et al., 2013). In the primate brain, rAAV5 outperformed all other serotypes by transducing significantly more cells in terms of transduction efficiency (Markakis et al., 2010). Furthermore, rAAV5 effectively transduced neurons as well as glial cells, whereas rAAV2 had a greater tropism for neurons (Davidson et al., 2000; Markakis et al., 2010). However, a comprehensive investigation of the impact of rAAV5 transduction on molecular and behavioral alterations has not been conducted yet.

The hippocampus is critical for memory formation, particularly declarative memories, and an impairment of the hippocampus can result in amnesia (Fanselow and Dong, 2010; Bannerman et al., 2014). In addition, the hippocampus has been traditionally linked to various emotional processes and, in particular, to anxiety (Fanselow and Dong, 2010; Bannerman et al., 2014). In this study, we generated three different doses of rAAV5 with a mChenry reporter gene and injected those vectors into the mouse hippocampus. mChenry^+^ cells were sorted for unbiased transcriptomic analysis. We further investigated glial responses and assessed the behavioral outcomes following rAAV5 transduction using three different doses, ultimately determining the optimal dosages of rAAV5 for preclinical trials targeting the hippocampus.

## Materials and methods

### Animals

Male CaMkIIα-cre mice (Jax lab, stock number 028867) were housed in standard laboratory cages with four to five mice per cage. The mice were kept on a 12-h light/dark cycle with lights on at 8:00 a.m. and in a temperature-controlled room with a range of 21–25°C. At the time of rAAV infusion, the mice were between 7 and 8 weeks old. *Ad libitum* food and water were provided. All animal procedures were approved by the Southern Medical University Animal Ethics Committee and conducted in compliance with the guidelines of the Chinese Council on Animal Care to minimize animal suffering and reduce the number of animals used.

### AAV vector production

The rAAV vector AAV5-EF1α-DIO-mCherry-WPRE (hereinafter referred to as rAAV5-mCherry) was packaged, purified, and tittered by Sunbio Medical Biotechnology (Shanghai, China). The double floxed inverted open-reading-frame (DIO) strategy makes mCherry expression in a Cre-responsive manner (Atasoy et al., 2008). The titter of rAAV5-mCherry was determined by real-time PCR as previously described (Rohr et al., 2002). Briefly, primers were designed to amplify the WPRE. The forward and reverse primers were 5′-CCTTTCCGGGACTTTCGCTTT-3′ and 5′-GACTAATACGTAGATGTACTGCCAAGTAGG-3′, respectively. Real-time PCR was carried out using an ABI 7500 Real Time PCR System (Applied Biosystems, USA). The PCR program was as follows: 95°C for 10 min followed by 40 cycles of 95°C for 10 s and 60°C for 35 s. AAV genome equivalents ranging from 10^4^ to 10^7^ were used to set up a DNA plasmid standard curve. The average titter of rAAV5-mCherry was 5.77 × 10^13^ vector genomes (v.g.)/ml. Serial dilutions were made to obtain the following doses: 2.88 × 10^8^, 2.88 × 10^9^, and 2.88 × 10^10^ v.g. per 0.5-μl (Ortinski et al., 2010).

### Virus injection

Mice were anesthetized with 1% pentobarbital sodium for all surgeries and placed in a stereotaxic frame (RWD, CN), which was conducted under aseptic conditions and guided by stereotaxic techniques. The skull was exposed, and four holes were drilled using a small steel burr at the following coordinates: −2 mm anteroposterior (AP), ± 1.6 mm mediolateral (ML), and −1.5 mm dorsoventral (DV) (dorsal hippocampus), and −2.9 mm AP, ±3.7 mm ML, and −3.8 mm DV (ventral hippocampus). All coordinates were relative to bregma. A 5 μl Hamilton syringe with a microinjector pump (KDS, Stoelting, USA) was used to inject the virus into the bilateral dorsal and ventral hippocampi at a rate of 0.05 μl/min, and the total volume of the 4 injections was 2 μl, with 0.5 μl being injected into each site. Once the injection was finished, the needle was lifted by 0.1 mm and left in position for an additional 10 min to facilitate virus diffusion at the injection site before being gradually removed. There were 15 mice in each group. Immunofluorescent staining, cell sorting experiments, and behavioral tests were conducted 3 weeks after virus injections when transgene expression reached asymptotic levels (Klein et al., 2006).

### Fluorescence-activated cell sorting

To isolate mCherry-labeled pyramidal neurons, we employed a modified version of a previously reported procedure (Brewer and Torricelli, 2007; Hempel et al., 2007; Zeisel et al., 2018). Mice were anesthetized with 1% pentobarbital sodium and then perfused through the left ventricle with ice-cold artificial cerebrospinal fluid (ACSF). ACSF consisting of 20 mM glucose, 75 mM sucrose, 1.25 mM NaH_2_PO_4_, 2.5 mM KCl, 26 mM NaHCO_3_, 87 mM NaCl, 7 mM MgSO_4_, 1 mM CaCl2, and 0.1 μM tetrodotoxin was equilibrated in 95% O_2_/5% CO_2_ before use. Tetrodotoxin (0.1 μM) was added to ACSF to inhibit neuronal action potentials and improve cell viability. The brain was removed, and 300 μm sections were collected in ice-cold ACSF using a Vibroslice microtome (VT 1000S; Leica). The hippocampus was microdissected and transferred to a 100-mm dish containing 7 ml digestion solution (2 mg/ml papain (Worthington, LS003119), 0.5 mM EDTA-2Na, and 2.5 ml/L Glutamax 100X (ThermoFisher) in Hibernate A (ThermoFisher, A1247501) and incubated for 30–40 min for enzymatic digestion at 30°C. The pH of the digestion solution was adjusted to 7.4 using neurobasal A (ThermoFisher, 10888022). After incubation, the tissue was delicately moved to a 15 ml tube filled with 5 ml ice-cold HABG [60 ml Hibernate A; 20 ml/L B_27_ 50X, (ThermoFisher), and 2.5 ml/L Glutamax 100X]. The tissue pieces were gently triturated through Pasteur pipettes with polished tip openings of 600, 300, and 150 μm diameter. The supernatant was collected in a 15 ml tube after settling. The sediment was resuspended in 2 ml HABG, and the supernatant after settling was transferred. These procedures were repeated until the sediment was dissociated. The cells were enriched by centrifugation (800 × g, 5 min, 4°C), resuspended in 1 ml HABG containing 1 mg/ml DNase I (Roche, 11284932001), and filtered with a 40 μm cell strainer (Falcon). To label dead cells, 0.1 μg/ml DAPI (Invitrogen) was added to the single-cell suspension. Then, the cells were immediately loaded into the Beckman MoFlo XDP Cell Sorter system for fluorescence-activated cell sorting (FACS). Cells with high mCherry and low DAPI fluorescence were isolated in 1.5 ml tubes containing 5.4 μl sample buffer, 0.6 μl RNase inhibitor, and 4 μl RNase-free water (Discover-sc WTA Kit V2, N711, Vazyme). For cell-sorting experiments, individual biological samples were produced by isolating 500–1,000 cells from 2 to 3 mice. For each group, 3–5 biological replicates were analyzed according to previous reports (Cahoy et al., 2008; Farmer et al., 2016; Wheeler et al., 2023).

### cDNA amplification and library construction

We used the Discover-scTM WTA Kit V2 for full-length poly(A)-RNA reverse transcription and cDNA amplification. All steps were carried out according to the manufacturer's instructions. Two controls were used: 1 ng standard total RNA was used as a positive control, and sheath fluid was used as a negative control to monitor the specificity of amplification and the possibility of contamination. The cDNA concentration was quantified by a Qubit 2.0 Fluorometer (Invitrogen, Carlsbad, CA, USA) and Agilent 2100 Bioanalyzer (Agilent Technologies, Santa Clara, CA, USA). Samples with a fragment distribution between 400 and 10,000 bp, a peak location of ~2,000 bp, and a total content of more than 2 ng were used for sequencing. After generating cDNA (1 ng), sequencing libraries were constructed using the TruePrepTM DNA Library Prep Kit V2 for Illumina^®^ (Vazyme, #TD503).

### RNA sequence

RNA sequencing was conducted by GENEWIZ (Suzhou, China), and the libraries were constructed and sequenced on an Illumina HiSeq X Ten instrument, following the manufacturer's instructions for 2 × 150 bp paired-end sequencing (Illumina, San Diego, CA, USA).

### Immunofluorescent staining

The procedures were modified from the previous reports (Chen et al., 2021). Mice were anesthetized with 1% pentobarbital sodium and intracardially perfused with saline followed by 4% polyformaldehyde. The brains were removed, postfixed in 4% polyformaldehyde for 12 h, and then equilibrated in 30% sucrose. Coronal sections of 40-μm thickness containing the hippocampus were collected. Brain slices were blocked for 1 h with 5% bovine serum albumin and 1% Triton X-100 at room temperature and then incubated with primary antibodies (rabbit anti-NeuN 1:300, Cell Signaling Technology, Cat# 12943; mouse anti-GFAP 1:500, Cell Signaling Technology, Cat# 3670 and rabbit anti-Iba1 1:500, Cat# 01919741) overnight at 4°C. The next day, the slices were incubated with secondary antibodies (Alexa 488-conjugated goat anti-rabbit and Alexa 647-conjugated goat anti-mouse, 1:500, Invitrogen) for 2 h at room temperature after washing three times with PBS and then incubated with DAPI (Invitrogen) for 10 min and washed for another three times. The slices were mounted on microscope slides. Fluorescence images were captured using a confocal microscope (A1R, Nikon). Cell counting and fluorescent area calculation were performed by ImageJ.

### Droplet digital PCR

Droplet digital PCR was conducted using the QX200 ddPCR EvaGreen Supermix kit (Bio-Rad, Pleasanton, CA). The sample concentrations were adjusted to 0.2 ng/μl. The total reaction volume was 20 μl, including 100 nM forward primer and 100 nM reverse primer for the target gene (Table 1), 10 μl ddPCR EvaGreen Supermix, cDNA template, and double distilled water. Three biological replicates were analyzed for each group. The 20-μl reaction mixture was loaded with 70 μl droplet generation oil (Bio-Rad, USA) into the wells in the middle row of a DG8™ cartridge to generate the droplet using the QX200 Droplet Generator. After processing, the droplets were transferred to the top wells of a 96-well PCR plate. PCR amplification was conducted on a T100 thermal cycler (Bio-Rad, USA) using a protocol as follows: 1 cycle of 95°C for 5 min, 40 cycles of 95°C for 30 s and 60°C for 60 s, 1 cycle of 4°C for 5 min, 1 cycle of 90°C for 5 min, and a hold at 4°C. The ddPCR data were analyzed with QuantaSoft™ analysis software. Positive droplets containing amplification products were discriminated from negative droplets by applying a fluorescence amplitude threshold, which was set to the highest value of the no template control (NTC) in QuantaSoft software.

**Table 1:** Primer sequences used for ddPCR analysis.

**Gene name**	**Forward primer**	**Reverse primer**
mCherry	CACTACGACGCTGAGGTCAA	GTAGTCCTCGTTGTGGGAGG
Gap43	ACCACCATGCTGTGCTGTAT	CTTCCGGCTTGACACCATCT
Camkv	GGTTATCGCTTCCTGCCTGT	GGCCCGGAAGATTTCACAGA
B2m	TTCTGGTGCTTGTCTCACTGA	CAGTATGTTCGGCTTCCCATTC
Cpne6	CGCATCCATGTGTGCTACTCAAAC	CAAAGGCTGCTTCTCTTCAAAAAAGT
Cplx1	ATTCAGAATGCCCTCTGCCC	CCCAGGTGAGCAAGGTACTG
Fos	TACTACCATTCCCCAGCCGA	GCTGTCACCGTGGGGATAAA

### RNA sequence data analysis

To ensure high-quality data and eliminate technical artifacts, such as adapters, PCR primers, fragments, and low-quality reads (with a Phred score < 20), raw data in fastq format were subjected to processing with Trimmomatic (v 0.30, RRID: SCR_011848) (Bolger et al., 2014). ***FastQC*** (v 0.11.8, RRID: SCR_014583) was used to determine sequencing quality. All libraries were aligned to UCSC mm10 using ***Hisat2*** (v 2.0.1, RRID:SCR_015530) (Kim et al., 2015). The clean data were monitored based on quality control (QC) criteria. QC-qualified samples from each group were included (Q30 > 85%, total mapped > 70%). Aligned reads were used for quantification by ***HTSeq*** (v0.6.1, RRID:SCR_005514). Fragments per kilobase of exon model per million reads mapped (FPKM) is used for the estimation of gene expression based on the read count. The analysis procedure is described below. To reduce noise, genes obeying read counts above 10 in each biological sample were included in the analysis. Differential gene expression was analyzed across all three groups by the likelihood ratio test (LRT) in the ***DESeq2*** (RRID:SCR_015687) Bioconductor package in the R (3.6.0) environment (RRID:SCR_001905) and an adjusted *p*-value of < 0.05 was used to indicate differential expression among the three groups (Love et al., 2014). Short time-series expression miner (STEM, v1.3.11, RRID:SCR_005016), a clustering method, was used to define a set of genes with similar expression patterns (Ernst and Bar-Joseph, 2006). We used this method to cluster differentially expressed genes (DEGs). DEG subsets with the same expression pattern, as determined by the STEM method, were subjected to functional enrichment analysis using the R package ***clusterProfiler*** (v 3.10.1, RRID:SCR_016884) (Yu et al., 2012), and a *p*-value of < 0.05 was considered to indicate significant enrichments. Heatmaps were constructed by using the complexHeatmap R package (RRID:SCR_017270) (Gu et al., 2016). Principal component analysis was conducted by using the plotPCA function in the R (3.6.0) environment.

### Mouse behavioral tests

A 3-day acclimation period was provided for all mice before conducting the behavioral assays, with each mouse handled for 5 min per day to minimize stress due to contact with the experimenter. In addition, mice were allowed to adjust to the testing room for 1 h before the experiments. All the experiments were performed from 13:00 to 18:00 unless otherwise indicated. Two different batches of mice were used for the experiment with the following sequence: OFT, EPM, fear conditioning test; Y maze test, NOR. Experiments were separated by at least 4 days when the same groups of mice were used in different behavioral tests. Behavioral tests were conducted as per the previous reports (Tian et al., 2017; Chen et al., 2021). There were 11–12 mice for each group.

#### The open field test

A plastic open-field chamber measuring 50 × 50 cm was used for the experiment, divided into a central field (25 × 25 cm) and an outer field (periphery). Individual mice were initially placed in the periphery of the field at the beginning of the 5 min test session, during which various parameters were measured, including average speed, the time spent in the center zone, and travel distance. The arena was cleaned with 70% ethanol between trials.

#### The elevated plus maze test

The elevated plus maze (EPM) comprises two open arms (10 × 50 cm) and two enclosed arms (10 × 50 × 40 cm) arranged in a plus-shaped configuration around a central platform (10 × 10 cm). The maze was elevated 40 cm above the ground, with arms of the same type facing each other. During the 5 min session of the EPM test, individual mice were placed in the center of the maze with their head facing a closed arm. The time spent in the open and closed arms, as well as the number of entries into the open arms, were quantified. The maze was wiped clean between trials with 70% ethanol.

Both the EPM and OFT sessions were recorded using a video camera, and the EthoVision XT video tracking system from Noldus in Wageningen, Netherlands, was employed to monitor the location, velocity, and movement of the mouse's head, body, and tail. All measurements were normalized to the mouse's body size and displayed accordingly.

#### Y-maze test

The Y-maze apparatus utilized in this experiment was composed of three gray acrylic arms (7.5 × 30 cm) joined together by a triangular neutral zone (7.5 × 7.5 × 7.5 cm) and enclosed by transparent acrylic walls (15 cm in height). Mice were initially placed in the central zone of the Y-maze, and their arm entries were observed and recorded over a period of 5 min. Alternation behavior, defined as consecutive entries into all three arms without repeating any, was calculated as a percentage of the total arm entries. To maintain cleanliness, the Y-maze was cleaned with a 70% ethanol solution between trials.

#### Novel object recognition test

On day 1, the mice were given 10 min to explore two identical objects that were placed in a 30 × 50 cm arena. After the exploration period, they were returned to their home cage. The next day, the mice were tested again in the same arena, but one of the previously seen objects was replaced with a new one, and they were given 5 min to explore. The discrimination index was calculated as (time spent exploring the novel object—time spent exploring the familiar object)/(total exploration time of both objects). To avoid discrimination of the objects based on odor, both the arena and the objects were thoroughly cleaned with 70% ethanol before and after each trial.

#### Fear conditioning test

Mice were trained and tested using the FreezeFrame system (Coulbourn Instruments). For fear conditioning, mice were habituated for 2 min on a shock grid (cage setup A: metal fence floor, gray metal wall) and then subjected to four fear conditioning pairs of a 30 s conditioned stimulus (CS) (tone at 75 dB, 2,800 Hz) and foot shock (1 s, 0.75 mA) at the end of the CS. Inter-tone intervals were randomly set between 60 and 120 s. For the contextual recall test, mice were placed in the training context for 3 min. For the tone-cued recall test, mice were placed in a different context (cage setup B: white Plexiglas floor, white Plexiglas wall) for an initial 2-min period, and this was followed by tone presentation for 3 min. The freezing behavior of mice was recorded by an infrared camera and subsequently analyzed using Coulborn-PC software. The chambers were wiped clean between trials with 70 % ethanol.

### Measurement of Il-1β and Csf1

The hippocampal tissue samples were collected from the animals 3 weeks after virus injection, homogenized, and centrifuged to obtain the supernatant. The protein concentration in the supernatant was determined using a BCA protein assay kit.

The concentrations of Il-1β and Csf1 were determined using ELISA kits obtained from Elabscience Biotechnology Co., Ltd. (E-EL-M0037c and E-EL-M2445c). The manufacturers' protocols were followed carefully to ensure that accurate measurements were obtained for each of the proteins of interest.

### Statistics

The experimental groups were not randomly assigned, and the ideal sample size was not predetermined. The normality of data distribution was assessed by the Shapiro–Wilk test. Statistical analysis of normally distributed data was performed using one-way ANOVA, followed by either the *post-hoc* Tukey's multiple comparison test or *post-hoc* Bonferroni analysis. Data were presented as mean ± SEM. Significance levels were denoted as ^*^P < 0.05, ^**^P < 0.01, and ^***^P < 0.001. The statistical software used for analysis was SPSS.

## Results

### Transduction efficiency of the three doses of rAAV5-mCherry

**Figure 1:**
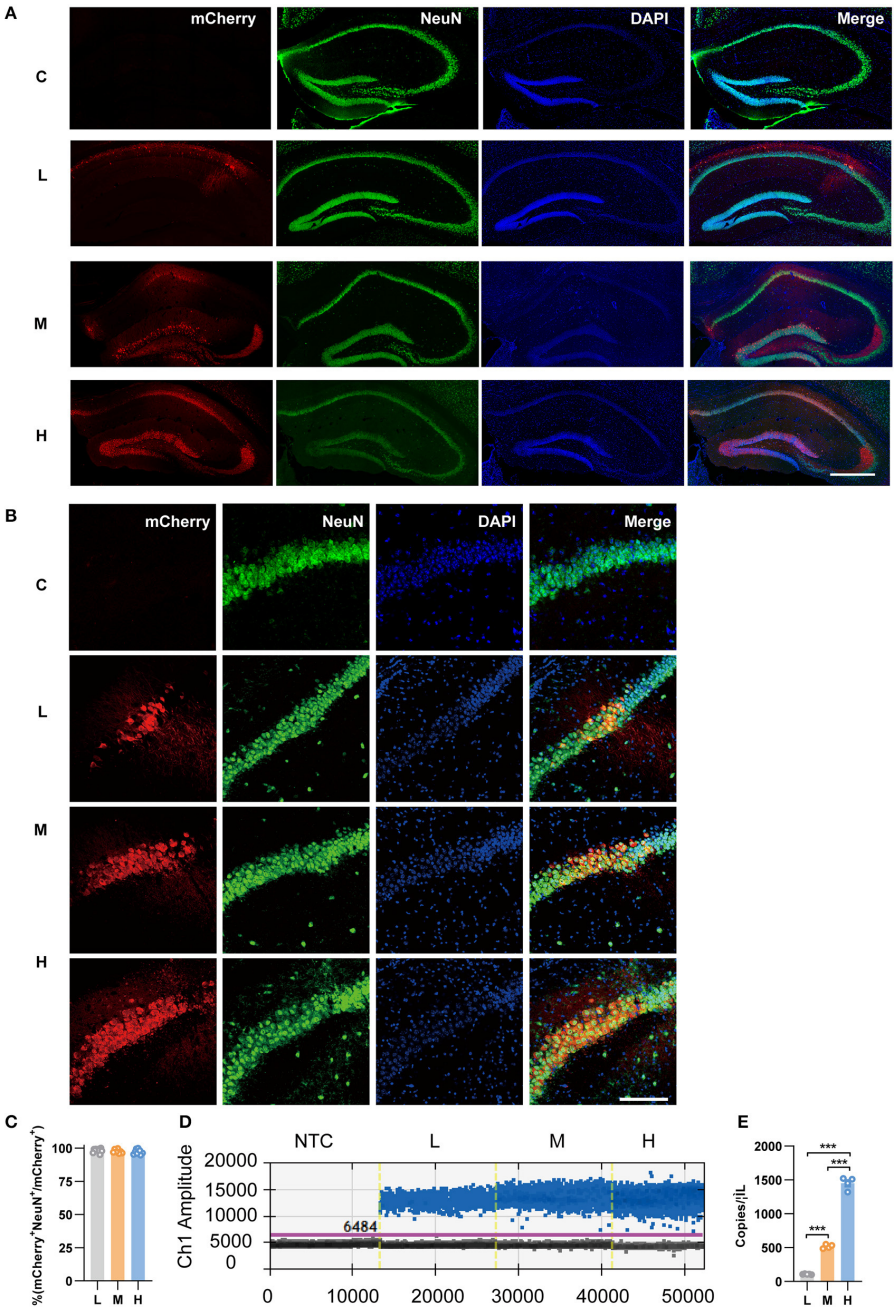
Transduction efficiency of three doses of rAAV5-mCherry in the hippocampi of CamkIIα-cre mice. **(A)** Representative image showed the transduction of the three doses of rAAV5-mCherry in hippocampal neurons. Scale bar, 500 μm. **(B)** Representative image showed most mCherry-positive cells colocalized with NeuN-positive cells. Scale bar, 100 μm. **(C)** Calculation data from **(B)**. **(D)** Representative fluorescence amplitude plot of mRNA expression in hippocampal pyramidal neurons among groups treated with the three different doses. Blue dots indicate the presence of the target gene sequence in the droplet, and gray dots indicate the absence of the sequence. **(E)** Calculation data from **(D)**. NTC, no template control; C, negative control; L, 2.88 × 10^8^ v.g.; M, 2.88 × 10^9^ v.g.; H, 2.88 × 10^10^ v.g. The data are presented as the mean ± S.E.M. ***P < 0.001.

To specifically express mCherry in pyramidal neurons, we infused the Cre-dependent rAAV vector rAAV5-EF1α-DIO-mCherry (abbreviated as rAAV5-mCherry) into the bilateral hippocampus of CamkIIα-Cre mice, which expressed the Cre recombinase under the pyramidal neuronal specific promoter CamkIIα. Three different doses were employed, among which 2.88 × 10^8^ genome copies per injection were defined as low dose, 2.88 × 10^9^ as medium dose, and 2.88 × 10^10^ as high dose according to previous reports (Ortinski et al., 2010). The morphology of the cell bodies expressing mCherry looked like neurons, and the colocalization of mCherry with the neuronal marker NeuN (Figures 1A–C) suggested specific expression of neurons by the virus. Consistent with previous research (Shemiakina et al., 2012), our data showed that pyramidal neurons expressing mCherry expressed apparent fluorescent puncta (Supplementary Figure 1), which are thought to be FP aggregates and precipitates in the cell (Yanushevich et al., 2002). The transduced volumes were dose-dependent, as the dose increased, neurons were efficiently transduced in CA3 and DG with rAAV5- mCherry in addition to CA1 (Figure 1A). We also evaluated mCherry expression levels by FACS-ddPCR. First, the hippocampal slices were sectioned 3 weeks after rAAV5-mCherry injection, and single-cell suspension was generated. Subsequently, mCherry-positive cells were isolated by FACS (Supplementary Figure 2A). Then, full-length poly(A)-RNA was reverse transcribed into cDNA and amplified with high quality cDNA (Supplementary Figure 2B). ddPCR results showed that the expression of mCherry increased with increasing dose of rAAV5- mCherry [Figures 1D, E; one-way ANOVA followed by *post-hoc* Tukey's multiple comparisons test, *F*_(2, 10)_ = 728.9, *p* < 0.0001; L vs. M, *p* < 0.0001; L vs. H, *p* < 0.0001; M vs. H, *p* < 0.0001].

### Transcriptomes of the three doses of rAAV5-mCherry

**Figure 2:**
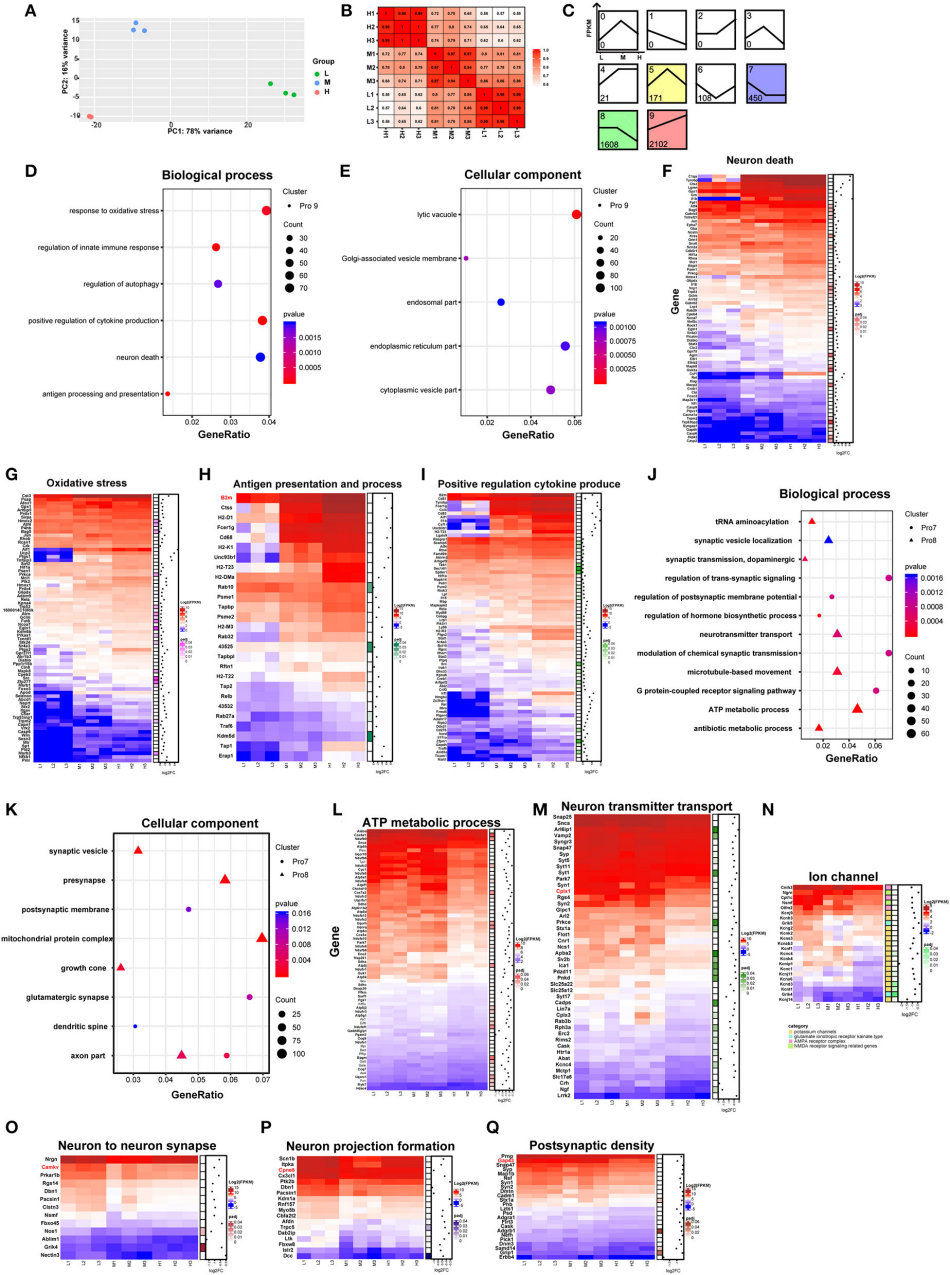
Comparison of neuronal transcriptomes among three different doses of rAAV5 transduction. **(A, B)** PCA plot and Pearson's correlation matrix of the groups treated with the three different doses based on gene read counts above 10. **(C)** STEM analysis of the profiles of genes with different expression patterns. **(D, E)** GO analysis of profile nine genes in hippocampal pyramidal neurons. The size of the circle corresponds to the number of significant genes whereas the color indicates the significance of the regional enrichment based on the normalized enrichment score. **(F–I)** FPKM heatmaps of core enrichment genes in profile 9. Blue, relatively low expression; red, relatively high expression. **(J, K)** GO analysis of profile 8 and profile 7 in hippocampal pyramidal neurons. **(L–Q)** FPKM heatmaps of core enrichment genes in profile 8 and profile 7. L, 2.88 × 10^8^ v.g.; M, 2.88 × 10^9^ v.g.; H, 2.88 × 10^10^ v.g.

To explore the effects of rAAV5-mCherry expression on neurons without bias, we generated RNA sequencing (NA-seq) data of the three different doses of rAAV5-mCherry. The low dose of rAAV5- mCherry was used as a default control. mCherry-positive cells were isolated by FACS followed by reverse transcription. High-quality samples were selected for fragment and library construction and sequenced by HiSeq X Ten. The analysis process is shown in Supplementary Figure 3. The reference genome (UCSC mm10) was used to map the clean data. Data accessibility and 12,345 genes with read counts above 10 in each sample were selected for follow-up analysis. Principal component analysis (PCA) and Pearson correlation matrix, both of which were based on FPKM values, demonstrated good reproducibility of samples within the groups (Figures 2A, B).

We analyzed the differentially expressed genes (DEGs) among the three groups by the likelihood rate test (LRT) using DESeq2. A total of 4,598 genes were found to be significantly different (*p* < 0.05) between groups, as shown in Supplementary Table 1. The software short time-series expression miner (STEM) was utilized to group genes exhibiting similar expression patterns based on their temporal changes, and a summary plot was generated to visualize the trends in gene expression. Using STEM analysis, we found that 2,102, 1,608, 450, 171, 108, and 21 DEGs were assigned to profile 9 (Pro 9), profile 8 (Pro 8), profile 7 (Pro 7), profile 5 (Pro 5), profile 6 (Pro 6), and profile 4 (Pro 4) (similarity ≥ 0.7 and *p* < 0.05), respectively, according to their expression patterns in the three groups (Figure 2C; Supplementary Table 2).

As the profile with the most genetic changes, the Pro 9 module was mainly mapped to the biological processes related to neuronal death and immune response (Figure 2D; Supplementary Table 3) and enriched in the following cellular components: the lysosome, the Golgi apparatus, the endoplasmic reticulum membrane, and the endosome according to the major gene ontology (GO) pathways analysis (Figure 2E; Supplementary Table 3). Notably, as the dose of rAAV5-mCherry increased, the expression of the genes related to neuron death, oxidative stress, antigen presentation and process, and positive regulation cytokine produce parallelly increased (Figures 2F–I). For example, genes involved in apoptotic programs, such as *Caspases 2, 6*, and *9* (Figure 2F); the antioxidative response-related gene *Hmox1, Tnfaip3*, and *Ucp2* (Richard et al., 2001; Su et al., 2017) (Figure 2G); the proinflammatory cytokines *Il-1*β*, Il-18* (Julien and Wells, 2017; Man et al., 2017), *colony-stimulating factor 1 (Csf1)* (Guan et al., 2016), and Ccl2 (Liu et al., 2013) (Figure 2F); the immune response-related gene *C1qa* (Yasuda et al., 2014) (Figure 2F); genes encoding proteins with MHC for antigen presentation and processing, such as *H2-DMa, H2-T22, B2m, H2-K1, H2-T23, H2-M3*, and *H2-D1* (Figure 2H); genes involved in inflammatory and infection processes or apoptosis, such as *Lgals9, Ccl4, Fcer1g, Stat1*, and *Cd83* (Kashio et al., 2003; Huang et al., 2005; Bellac et al., 2007; Szczucinski and Losy, 2007; Glezer et al., 2009) (Figure 2I), were significantly upregulated in high-dose groups.

The DEGs in Pro 8 were determined to be mainly involved in ATP metabolic processes and neurotransmitter transport and to be enriched in several cellular components, such as the mitochondrial protein complex, synaptic vesicles, distal axons, and the synaptic membrane (Figures 2J, K; Supplementary Table 3). In addition, the DEGs in Pro 7 were determined to be mainly involved in the postsynaptic membrane potential, neuronal projections, and apoptosis and to be enriched in actin filaments (Figures 2J, K; Supplementary Table 3). The enrichment analyses suggested that mitochondrial function, synaptic plasticity, and transmitter release are likely to be dampened in a dose-dependent manner (Figures 2L–Q). Specifically, genes encoding mitochondrial ribosome proteins (e.g., *Mrpl28, Mrpl37, Mrps11*, and *Mrps12*) and involved in ATP synthase (e.g., *Atp5a1, Atp5b, Atp5d*, and Atp5g1) were downregulated. Mitochondria are critical for ATP generation, programmed cell death, and signal transduction (Friedman and Nunnari, 2014), and mitochondrial DNA (mt-DNA) released from impaired mitochondria would trigger proinflammatory and type I interferon responses (West et al., 2015; West and Shadel, 2017). Our data also showed that mt-DNAs, such as *mt-Rnr2, mt-Nd1, mt-Nd2, mt-Co1, mt-Co2*, and *mt-Co3* in Pro9, were upregulated dose-dependently (Supplementary Figure 4). Taken together, these results suggest high-dose rAAV5-mCherry induced diminished mitochondrial function characterized by reduced ATP supply and proinflammation response.

Genes involved in synaptic plasticity, such as *Cplx1, Camkv, Cpne6*, and Cap43, were decreased (Figures 2O–Q) in a high-dose group. Among them, postsynaptic *Cplx1* regulates AMPA receptor delivery to synapses during long-term potentiation (LTP) (Ahmad et al., 2012), and LTP is widely considered one of the major cellular mechanisms that underlie learning and memory (Bliss and Collingridge, 1993). Mice with *Camkv* knockdown from the CA1 pyramidal neurons exhibited marked synaptic transmission and plasticity impairments (Liang et al., 2016). *Cpne6*, an isoform of copine that is expressed at extremely high levels in mouse hippocampal pyramidal neurons, is required for memory and synaptic plasticity as well as LTP (Reinhard et al., 2016). *Gap43*, a neuronal-specific protein in the postsynaptic density gene set, is required for proper learning and memory formation and neuronal repair after injury (Hulo et al., 2002; Rekart et al., 2005; Holahan and Routtenberg, 2008). Altogether, these findings suggest high-dose rAAV5-mCherry induced altered synaptic plasticity underlying learning and memory.

Last but not least, the genes in the Pro 6 module were enriched in the biological processes of protein acylation, postsynaptic organization, and regulation of the apoptotic signaling pathway. The genes in the Pro 5 module were mapped to the GO biological processes of the ERK1 and ERK2 cascade and negative regulation of proteolysis. The genes in the Pro 4 module were enriched in the negative regulation of immune system processes and regulation of myeloid cell differentiation (Supplementary Table 3).

### Neuronal and glial response to hippocampal transduction

**Figure 3:**
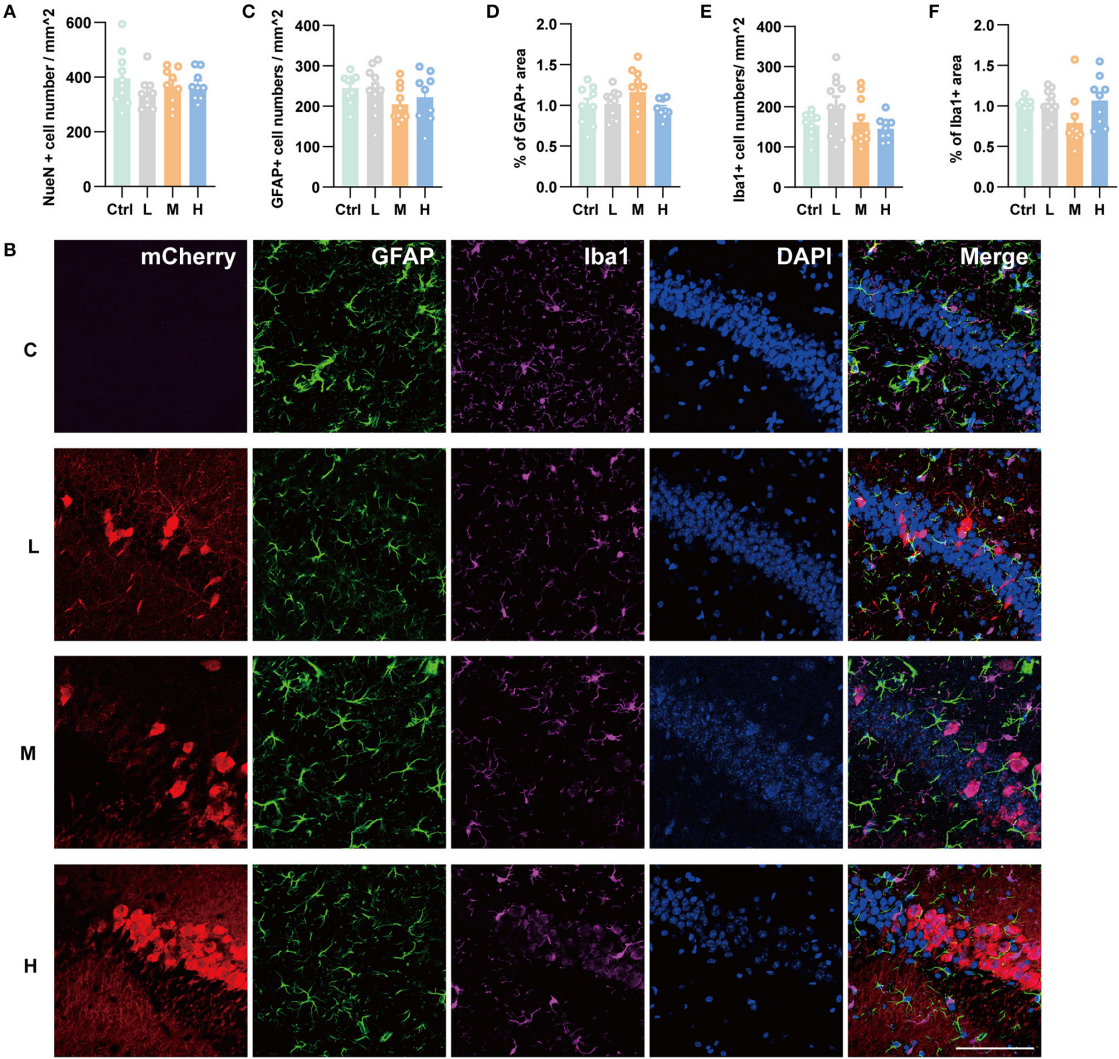
Immunohistochemical staining showed no hippocampal neuronal loss and gliosis after three different doses of rAAV5 transduction. **(A)** Statistics of NeuN^+^ cell count in the hippocampus. **(B)** Representative staining for astrocytes (GFAP^+^) and microglia (Iba1^+^) of low, medial, and high virus vector transduction. Scale bar, 100 μm. **(C)** Statistics of GFAP^+^ cell count in the hippocampus. **(D)** Statistics of the percentage of GFAP^+^ area in the hippocampus. **(E)** Statistics of Iba1^+^ cell count in the hippocampus. **(F)** Statistics of the percentage of Iba1^+^ area in the hippocampus. C, negative control; L, 2.88 × 10^8^ v.g.; M, 2.88 × 10^9^ v.g.; H, 2.88 × 10^10^ v.g. The data are presented as the mean ± S.E.M.

**Figure 4:**
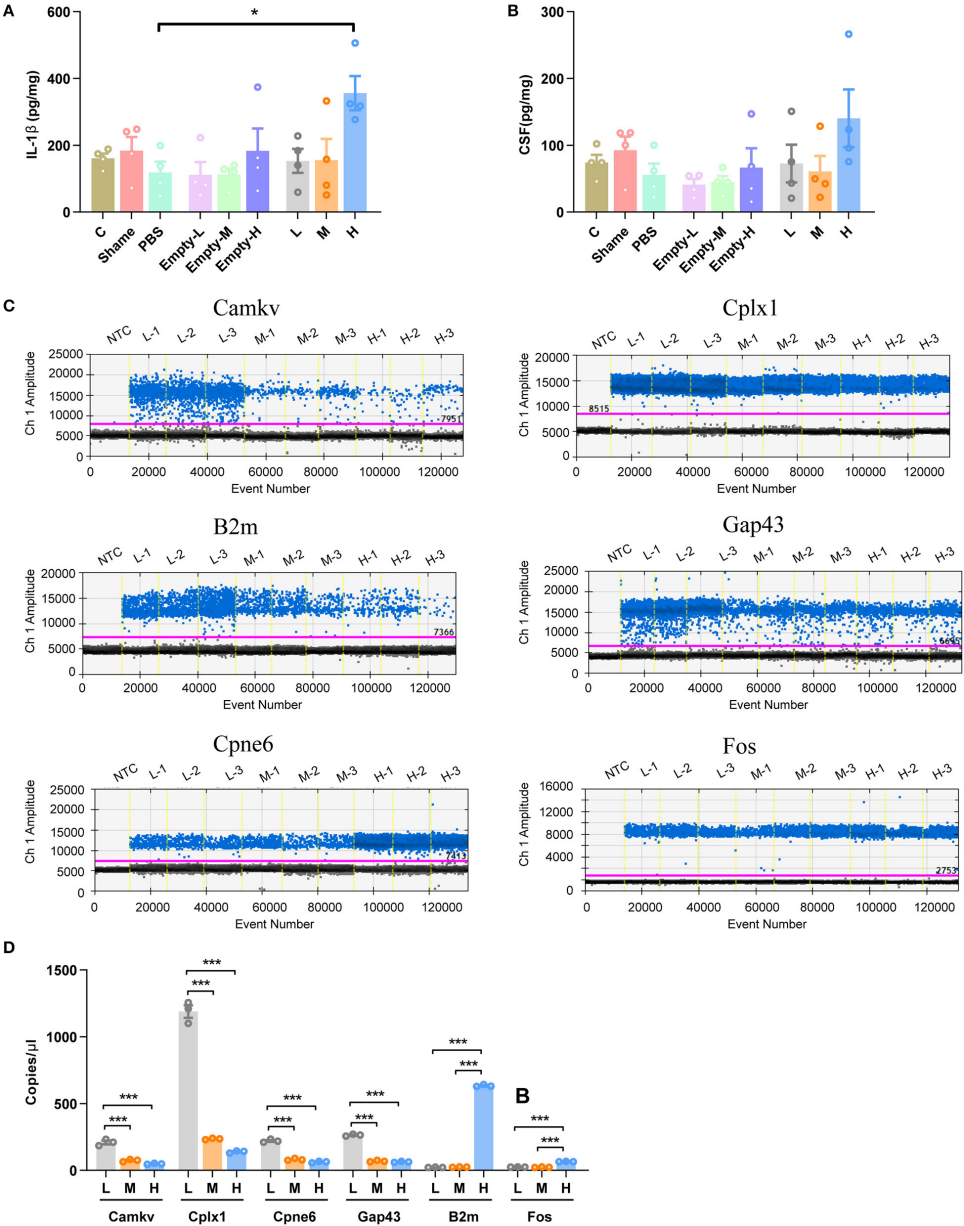
Validation of some DEG involved in immune response and synaptic plasticity. **(A)** The concentration of IL-1β. **(B)** The concentration of CSF1. **(C)** Representative fluorescence amplitude plot of *Camkv, Cplx1, Cpne6, Gap43, B2m*, and *Fos* mRNA expression in hippocampal pyramidal neurons. The blue dots indicate the presence of the target gene sequence in the droplet, and the gray dots indicate the absence of the sequence. **(D)** The expression of *Camkv, Cplx1, Cpne6*, and *Gap43* was significantly increased in medium-dose and high-dose treated mice compared with the low-dose group, while the expression of *B2m* and *Fos* was specifically upregulated in the high-dose group. *N* = 4 for each group in **(A, B)**, *N* = 3 for each group in **(C, D)**. NTC, no template control; C, negative control; Sham, non-injected group; Empty-L, 2.88 × 10^8^ v.g. non-coding AAV-injected group; Empty-M, 2.88 × 10^9^ v.g. non-coding AAV-injected group; Empty-H, 2.88 × 10^10^ v.g. non-coding AAV-injected group; L, 2.88 × 10^8^ v.g.; M, 2.88 × 10^9^ v.g.; H, 2.88 × 10^10^ v.g. The data are presented as the mean ± S.E.M. *P < 0.05, and ***P < 0.001.

As genes involved in neuronal death and oxidative stress upregulated parallelly with a dose of rAAV5 increased, we then performed immunohistochemistry staining neuronal marker NeuN to compare the cellular alterations in the hippocampus of mice transduced with the three different doses of rAAV5-mCherry. We did not observe any significant distortions in the distribution of hippocampal neurons or the structure of the hippocampus among the three groups (Figure 1A). NeuN^+^ quantification revealed that there is no neuronal loss as NeuN^+^ counting was comparable among groups [Figure 3A; one-way ANOVA, *F*_(3, 33)_ = 0.8890, *p* = 0.4570]. Both astrocytes and microglia respond to, and contribute to, neuroinflammatory cues in the CNS (Sofroniew, 2015; Wolf et al., 2017). To explore whether astrocytes and microglia were upregulated after virus injection, sections were stained for astrocytic marker glia fibrillary acidic protein (GFAP) and microglia marker Iba1 (Figure 3B). Quantification of GFAP^+^ cell number and GFAP^+^ area revealed that the medial and high- doses rAAV5-mCherry did not induce astrogliosis when compared with the low-dose rAAV5-mCherry [Figure 3C, one-way ANOVA, *F*_(3, 35)_ = 1.2890, *p* = 0.2935; Figure 3D, one-way ANOVA, *F*_(3, 35)_ = 1.7000, *p* = 0.1848]. Similarly, the quantification of Iba1^+^ cell number and Iba1^+^ area also revealed that the medial and high-doses of rAAV5-mCherry did not induce microgliosis when compared with the low-dose rAAV5-mCherry [Figure 3E, one-way ANOVA, *F*_(3, 35)_ = 2.4820, *p* = 0.0770; Figure 3F, one-way ANOVA, *F*_(3, 35)_ = 2.5760, *p* = 0.0649]. In a similar study, we found that more reactive astrocytes were induced by targeting transgene expression to astrocytes with high-dose rAAV5-mCherry in Aldh1l1-creER^T2^ mice than by targeting transgene expression to astrocytes with a low-dose group (Supplementary Figure 5). In addition, the proinflammatory cytokines, Il-1β and CSF, whose genes showed upregulation in GO analysis (Figure 2F) were tested. We found that a high dose of rAAV5-mCherry significantly increased the concentrations of Il-1β when compared with the buffer (PBS)-injected group [Figure 4A, one-way ANOVA, *F*_(8, 27)_ = 2.969, *P* = 0.0161, H vs. PBS: *P* = 0.0158], while had no effect on CSF [Figure 4B, one-way ANOVA, *F*_(8, 27)_ = 1.612, *P* = 0.1680], suggesting that high-dose of rAAV5-mCherry expression may induce an immune response.

We next validated six DEGs (*Cplx1, Camkv, Cpne6, Gap43, Fos*, and *B2m*) that are involved in synaptic plasticity underlying learning and memory (Figure 2). mCherry^+^ cells from three different doses of rAAV5-mCherry were sorted by FACS and proceeded for ddPCR. The expression of B2m and Fos were decreased specifically in the high-dose group, while *Camkv, Cplx, 1Cpne6*, and *Gap43* were upregulated in both medium-dose and high-dose groups compared with the low-dose group [Figures 4C, D, one-way ANOVA, Tukey's *post-hoc* multiple comparisons test; *Camkv*, *F*_(2, 6)_ = 103.0, *p* < 0.0001, M vs. L: *P* < 0.0001, H vs. L: *p* < 0.0001, H vs. M: *P* = 0.1800; *Cplx1*, *F*_(2, 6)_ = 464.7, *p* < 0.0001, M vs. L: *p* = 0.0001, H vs. L: *p* < 0.0001, H vs. M: *p* = 0.0970; *Cpne6*, *F*_(2, 6)_ = 279.4, *p* < 0.0001, M vs. L: *p* < 0.0001, H vs. L: < 0.0001, H vs. M: *p* = 0.1075; *Gap43*, *F*_(2, 6)_ = 1231, *p* < 0.0001, M v.s L: *p* < 0.0001, H vs. L: *p* < 0.0001, H vs. M: *p* = 0.4425; *B2m*, *F*_(2, 6)_ = 18005, *p* < 0.0001, M vs. L: *p* = 0.8557, H vs. L: *p* < 0.0001, H vs. M: *p* < 0.0001; *Fos*, *F*_(2, 6)_ = 625.5, *p* < 0.0001, M vs. L: *p* = 0.6148, H vs. L: *p* < 0.0001, H vs. M: *p* < 0.0001], suggesting that medium-dose and high-dose of rAAV5-mCherry expression may alter neuronal synaptic plasticity.

### High-dose rAAV5-mCherry injection impaired spatial working memory and contextual fear memory

**Figure 5:**
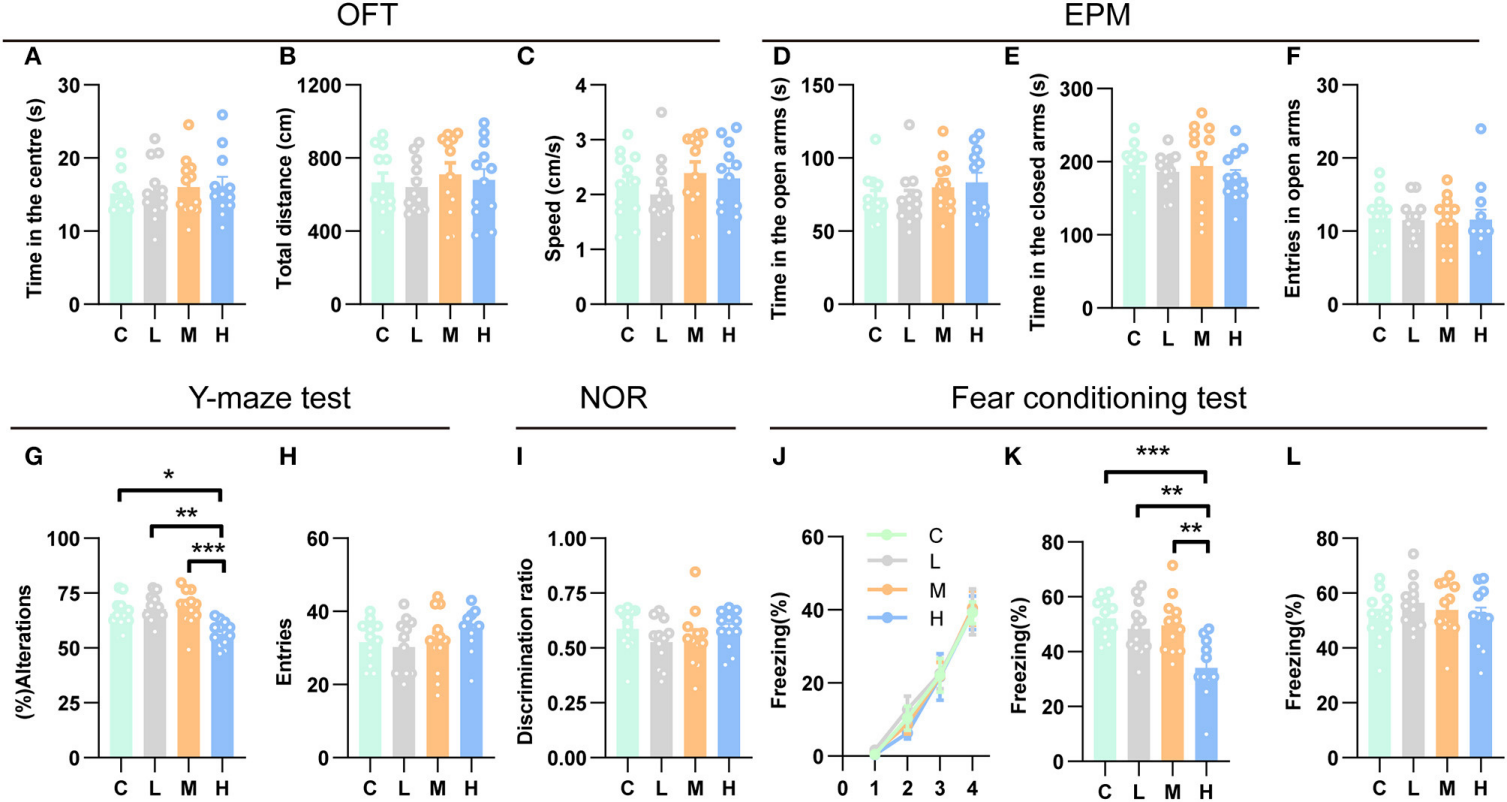
High-dose rAAV5-mCherry transduction impaired spatial working memory and contextual fear memory. **(A–F)** rAAV5-mCherry transduction had no effect on anxiety-related behaviors **(A, D, E, F)** and locomotion **(B, C)** in the OFT and EPM. **(G, H)** High-dose rAAV5-mCherry transduction impaired spatial working memory in the Y-maze task. **(I)** rAAV5-mCherry transduction had no effect on novel object recognition memory in the NOR. **(J–L)** High-dose rAAV5-mCherry transduction exhibited impaired contextual fear memory retrieval **(K)** without alteration in fear learning **(J)** and tone-cued memory retrieval **(L)**. C, negative control; L, 2.88 × 10^8^ v.g.; M, 2.88 × 10^9^ v.g.; H, 2.88 × 10^10^ v.g. The data are presented as the mean ± S.E.M. *P < 0.05, **P < 0.01, and ***P < 0.001.

Recent studies have indicated that the hippocampus may play a role in emotional behavior, as both human and animal research has linked hippocampal dysfunction to mood and anxiety disorders (Fanselow and Dong, 2010; Bannerman et al., 2014). To investigate whether different doses of rAAV5- mCherry anxiety-related behaviors, we employed two well-validated anxiety assays, the open field test (OFT) and the elevated plus maze test (EPM) (Carola et al., 2002). We first evaluated the effects of efficient gene transduction by rAAV5- mCherry on anxiety-related behaviors and locomotor activity in the OFT. Mice that were subjected to the OFT 3 weeks after rAAV5-mCherry injection with different doses spent similar time in the central area [Figure 5A, one-way ANOVA; *F*_(3, 44)_ = 0.1828, *p* = 0.9075]. Meanwhile, the rAAV5-mCherry expression did not affect the overall activity of the mice as indicated by total path length [Figure 5B, one-way ANOVA; *F*_(3, 44)_ = 0.2709, *p* = 0.8460] and speed [Figure 5C, one-way ANOVA; *F*_(3, 44)_ = 0.8349, *p* = 0.4819] in the OFT. Additionally, the rAAV5-mCherry expression had no effect on anxiety-related behaviors in the EPM either [Figures 5D–F, one-way ANOVA; D, *F*_(3, 44)_ = 0.9396, *p* = 0.4297; E, *F*_(3, 44)_ = 0.4489, *p* = 0.7124; F, *F*_(3, 44)_ = 0.0546, *p* = 0.9830].

Numerous studies have demonstrated an essential role for the hippocampus in several forms of memory formation (e.g., spatial learning, working memory, recognition memory, or contextual fear) (Fanselow and Dong, 2010; Bannerman et al., 2014). Our transcriptomes and ddPCR results showed that synaptic plasticity underlying learning and memory may get altered after medium-dose or high-dose of rAAV5-mCherry transduction, raising the question that whether medium-dose or high-dose of rAAV5-mCherry transduction would affect learning and memory. To this end, we employed a battery of learning and memory paradigms: the novel object recognition test, the contextual fear conditioning test, and the Y-maze spontaneous alternation test. In the Y-maze spontaneous alternation test, a decrease in the percentage of alternation was observed following high-dose rAAV5-mCherry transduction, while no change was found by medium- or low-dose rAAV5-mCherry transduction compared with the control group [Figure 5G, one-way ANOVA, Tukey's *post-hoc* multiple comparisons test; *F*_(3, 44)_ = 7.7960, *p* = 0.0003, C vs. L: *P* = 0.8656; C vs. M: *P* = 0.7313; C vs. H: *P* = 0.0115; L vs. M: *P* = 0.9939, L vs. H: *p* = 0.0012, M vs. H: *P* = 0.0006]. No significant difference was observed in the number of entries among the groups in the Y-maze spontaneous alternation test. [Figure 5H, one-way ANOVA; *F*_(3, 44)_ = 0.9231, *p* = 0.4376]. In contrast, in the object recognition test, medium- and high-dose rAAV5-mCherry transduction showed similar performance with the low-dose group [Figure 5I, one-way ANOVA; *F*_(3, 44)_ = 0.9615, *p* = 0.4194]. However, in the contextual fear conditioning test, high-dose rAAV5-mCherry transduction exhibited impaired contextual memory retrieval, while no impairment was found in the medium- or low-dose group compared with the control group [Figure 5J, one-way ANOVA, Tukey's *post-hoc* multiple comparisons test; *F*_(3, 43)_ = 8.0990, *p* = 0.0002, C vs. L: *P* = 0.7604; C vs. M: *P* = 0.9078; C vs. H: *P* = 0.0003; L vs. M: *P* = 0.9893, L vs. H: *p* = 0.0049, M vs. H: *P* = 0.0020], with similar fear learning [Figure 5K, two-way ANOVA; *F*_time × *group*(9, 129)_ = 0.1575, *p* = 0.9975] and tone-cued retrieval [Figure 5L, one-way ANOVA; *F*_(3, 43)_ = 0.8156, *p* = 0.4923] among groups.

## Discussion

In this study, we comprehensively analyzed the transcriptome, glial response, and behavior outcome of rAAV5-mCherry injection with three different doses in the mouse hippocampus to provide a detailed dataset that allows direct dose-comparisons and thus can be used to choose an optimal dose for gene delivery in preclinical trails.

For the first time, we performed the unbiased RNA sequence and include an in-depth analysis. High-dose rAAV5-mCherry (2.88 × 10^10^) significantly upregulated genes related to the immune response and apoptosis; meanwhile, downregulated genes associated with mitochondrial function and synaptic plasticity in hippocampal pyramidal neurons, suggesting that high-dose rAAV5-mCherry may enhance the fragility of hippocampal pyramidal neurons and impair synaptic plasticity. These findings agree with previous reports showing that high-dose rAAV5-gfa104-eGFP (3 × 10^10^) injection into the hippocampus generates deficits in neuronal inhibition (Ortinski et al., 2010). Of note, high-dose rAAV5-mCherry did not affect the distribution of hippocampal neurons and the structure of the hippocampus. In contrast, high-dose rAAV8-GFP (1 × 10^10^) transduction led to dopaminergic neuronal toxicity in the substantial nigra, resulting in a significant loss of dopamine neurons (Klein et al., 2006). In addition, subretinal injection of high-dose rAAV8-CMV-GFP (2 × 10^9^) caused almost complete renal pigment epithelium cell loss (Xiong et al., 2019). Different serotypes (rAAV5 vs. rAAV8), promoter (EF1α vs. CMV), transgene (mCherry vs. GFP), different cell types (pyramidal neurons vs. dopamine neurons vs. pigment epithelium cells) and regions (hippocampus vs. substantial nigra vs. retina) transduced may account for the differences in these studies.

While our results convincingly demonstrate that deleterious transcriptome changes are observed, the study lacks proper controls to understand what is driving the changes. An important question is whether this is inherent to AAV as a vector system. It is not clear which of the following is driving the transcriptome changes (or a combination of these, or something else): mCherry, either due to an immune response or mCherry protein itself; AAV (transgene independent); TLR9 response; injection trauma; transduction of astrocytes (without gene expression) causing secondary effects to neurons; and impurities in the AAV preparation. It is, therefore, critical to employ proper controls to elucidate the issue. For example, injections in wild-type mice of the same vector doses which would exclude Cre-dependent expression and empty vector would tease apart the effect of the viral elements on inflammation compared to the effect of expression of fluorescent protein. The problem is that it is technically difficult to implement as the transduced cells in these control groups are not tagged, and we cannot sort these cells for RNA-seq. Our ELISA experiments (Figures 4A, B) provided some implications: (1) the non-injected group (Sham), buffer-injected group (PBS), and low/medial/high-dose of the non-coding AAV-injected group (Empty-L, Empty-M, Empty-H) did not show any immune response compared with the naïve control group (C), suggesting that the injection trauma, buffer, and AAV vector itself did not contribute to the upregulated proinflammatory cytokines; (2) IL-1β increased in the high-dose of AAV-mCherry group (H) compared with PBS treatment but not between the high-dose of AAV-mCherry group and high-dose of the non-coding AAV-injected group, suggesting that the AAV vector itself and the transgene expression together contributed the changes of IL-1β. State-of-art technology, such as spatially resolved transcriptomics (Crosetto et al., 2015), can be used to solve this problem, which needs to be explored further.

Transduction of high-dose rAAV5-mCherry in hippocampal neurons did not induce gliosis in our study. While it has been reported that high-dose rAAV5-gfa104-eGFP (3 × 10^10^) injection into the hippocampal astrocytes significantly increased GFAP and vimentin expression in astrocytes, indicating astrocyte reactivity (Ortinski et al., 2010). Consistently, we found that astrocytic transduction of high-dose rAAV5-mCherry in the hippocampus induced reactive astrogliosis. Intracranial transduction of high-dose rAAV1 and rAAV9 (10^10^) elevated neutralizing antibody titers in the blood sera in rhesus monkeys (Mendoza et al., 2017).

Notably, hippocampal neuronal transduction of high-dose rAAV5-mCherry impaired working memory and contextual fear, without effects on locomotion and anxiety-related behaviors. As the hippocampus is primarily involved in the cognitive process of learning and memory and the core pathological brain region of AD (Lane et al., 2016), caution should be made when gene therapy strategies are employed for AD treatment.

In summary, by comparisons of the transcriptome, glial response, and behavior outcome of neuronal transduction with three different doses of virus vectors in the mouse hippocampus, our data reveal a maladaptive effect of high-dose vs. median-dose or low-dose with increased neural fragility and impaired memory. Therefore, in addition to considering the effective expression of exogenous genes, the possible side effects should be taken into account in clinics. However, the molecular mechanisms underlying these disadvantages of high-dose rAAV5-mCherry are yet to be studied in the future.

## Data availability statement

The datasets presented in this study can be found in online repositories. The names of the repository/repositories and accession number(s) can be found in the article/ Supplementary material.

## Ethics statement

The animal study was reviewed and approved by Southern Medical University Animal Ethics Committee.

## Author contributions

Y-HC and T-MG designed and supervised the studies and wrote the manuscript. Y-SL and M-LW performed the experiments with the help of N-YH, Z-ML, J-LW, and HL. Y-HC and Y-SL analyzed the data and generated the figures. X-WL provided critical consumables. Y-HC and T-MG interpreted the results with critical input from J-MY. All authors read and approved the manuscript.
