# Supplementary material for: A comparison of the impact on neuronal transcriptome and cognition of rAAV5 transduction with three different doses in the mouse hippocampus
Source: Front Mol Neurosci. 2023 Jul 14;16:1195327. doi: 10.3389/fnmol.2023.1195327 (PMC10375024; doi:10.3389/fnmol.2023.1195327)
Supplement: Supplementary file 4 [file Data_Sheet_1.pdf]

A Comparison of Impact on Neuronal Transcriptome and Cognition of rAAV5 Transduction with Three Different Does in the Mouse Hippocampus

Yi-Si Liu[1, †], Meng-Ling Wang[1, †], Neng-Yuan Hu[1], Zi-Ming Li[1], Jia-Li Wu[1], Hao Li[1], Jing-Ting Li[1], Xiao-Wen Li[1], Jian-Ming Yang[1], Tian-Ming Gao[1, *], Yi-Hua Chen[1, *]

1, State Key Laboratory of Organ Failure Research, Key Laboratory of Mental Health of the Ministry of Education, Guangdong-Hong Kong-Macao Greater Bay Area Center for Brain Science and Brain-Inspired Intelligence, Guangdong Province Key Laboratory of Psychiatric Disorders, Department of Neurobiology, School of Basic Medical Sciences, Southern Medical University, Guangzhou, China.

†, These authors contribute equally to this work and share first authorship.

*, Correspondence: chenyihua@smu.edu.cn (Y.H.C); tgao@smu.edu.cn (T.M.G)

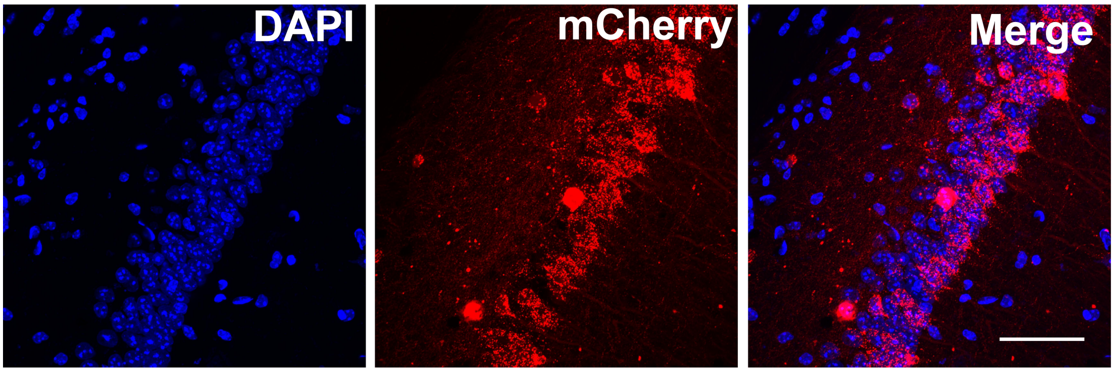

Supplementary Figure 1 The mCherry fluorescent punta in the cytoplasm of pyramidal neurons. Scale bar, 50 μm.

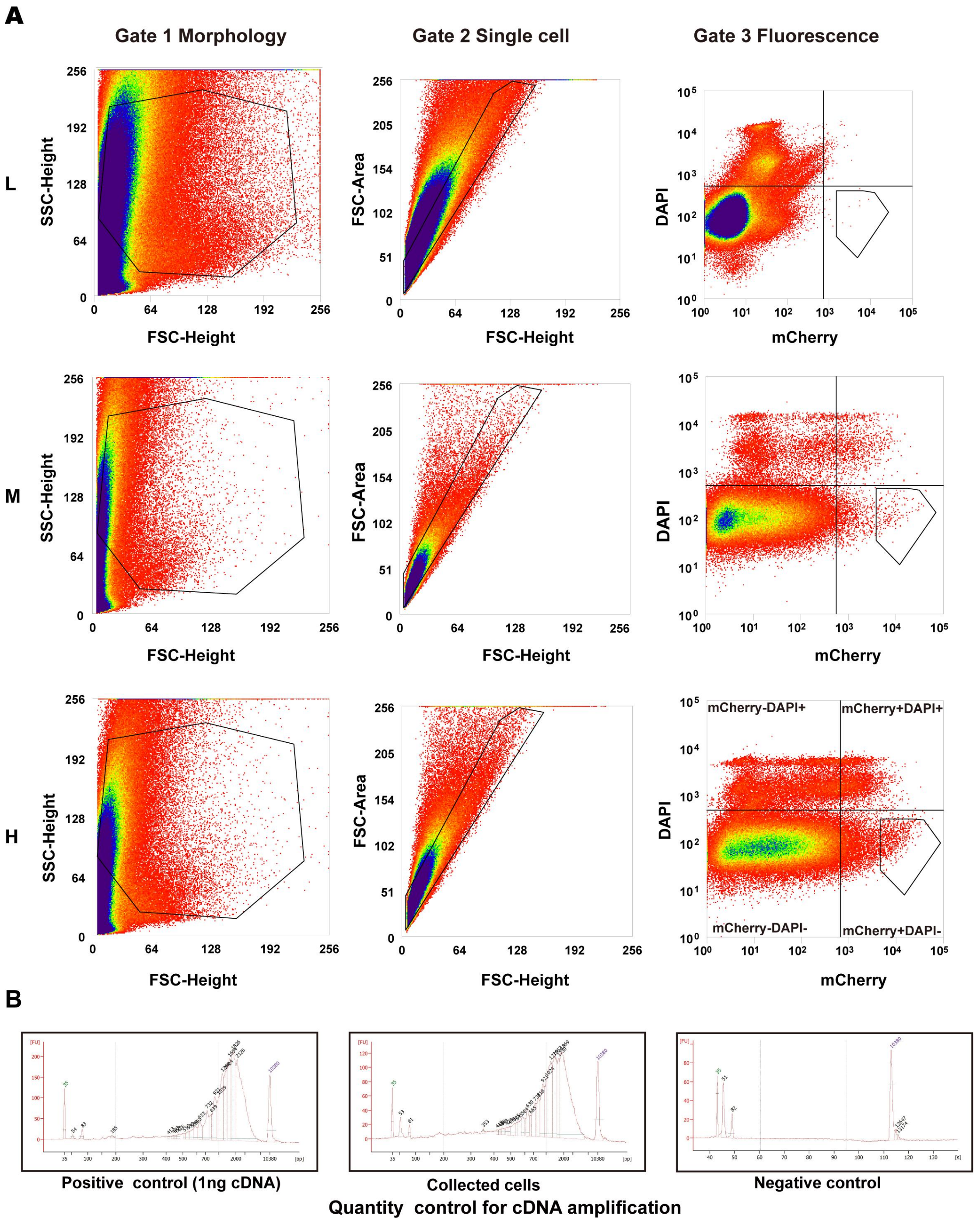

Supplementary Figure 2 A, Flow cytometric analysis of the groups treated with the three different doses. In the FACS plot of SSC height (granularity) versus FSC height (cell size), debris was found in the bottom left corner with lower forward scatter levels. Gate 1 was applied to exclude debris. The FSC-area vs. FSC-height plot was used to discriminate adhered cells from single cells. The doublet has the same height as one of them, but the area is 2x larger, corresponding to 2 cells, leading to inaccurate conclusions. Gate 2 was used to exclude adhered cells. The two-parameter density blot was used to distinguish dead cells from mCherry-positive cells by creating a plot of DAPI vs. mCherry. Gate 3 was used to select cells with strong mCherry and weak DAPI fluorescence. The positions of the three Gates were fixed,

and then the parameters of the flow cytometer were adjusted so that mCherry strong fluorescent and DAPI weakly fluorescent cells appeared in Gate 3 for each sample. Approximately 500 - 1,000 cells were collected for each sample. **B,** Electropherogram traces for amplified cDNA from the standard positive (1 ng RNA), collected cells and negative controls (sheath fluid) were generated on the Agilent 2100 Bioanalyzer. The cDNA should show a distinct peak that spans 400 to 10,000 bp with a peak at approximately 2,000 bp.

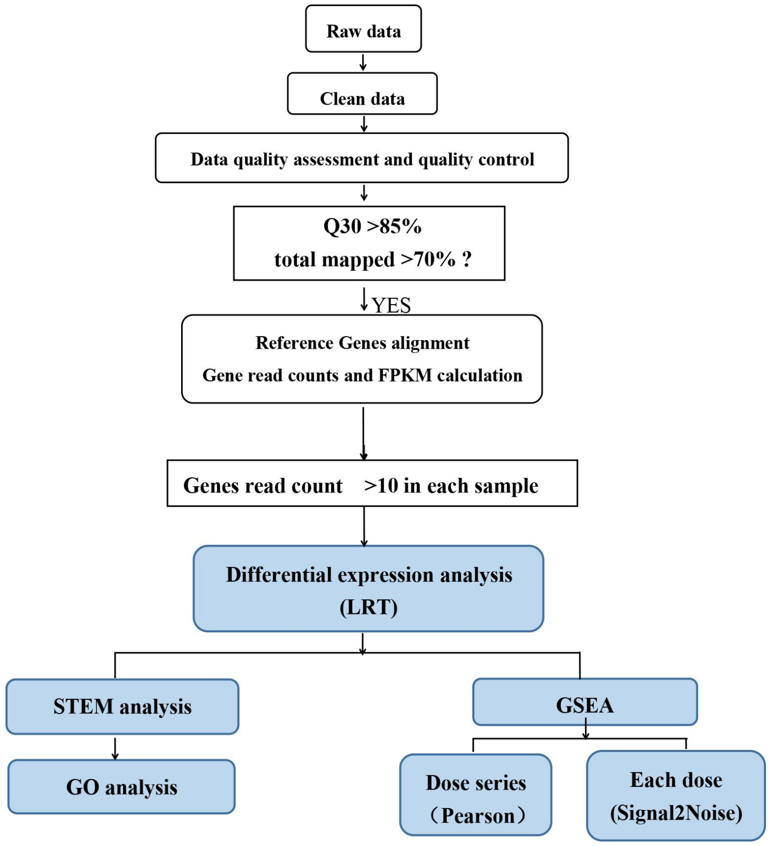

Supplementary Figure 3. The RNA - seq analysis process.

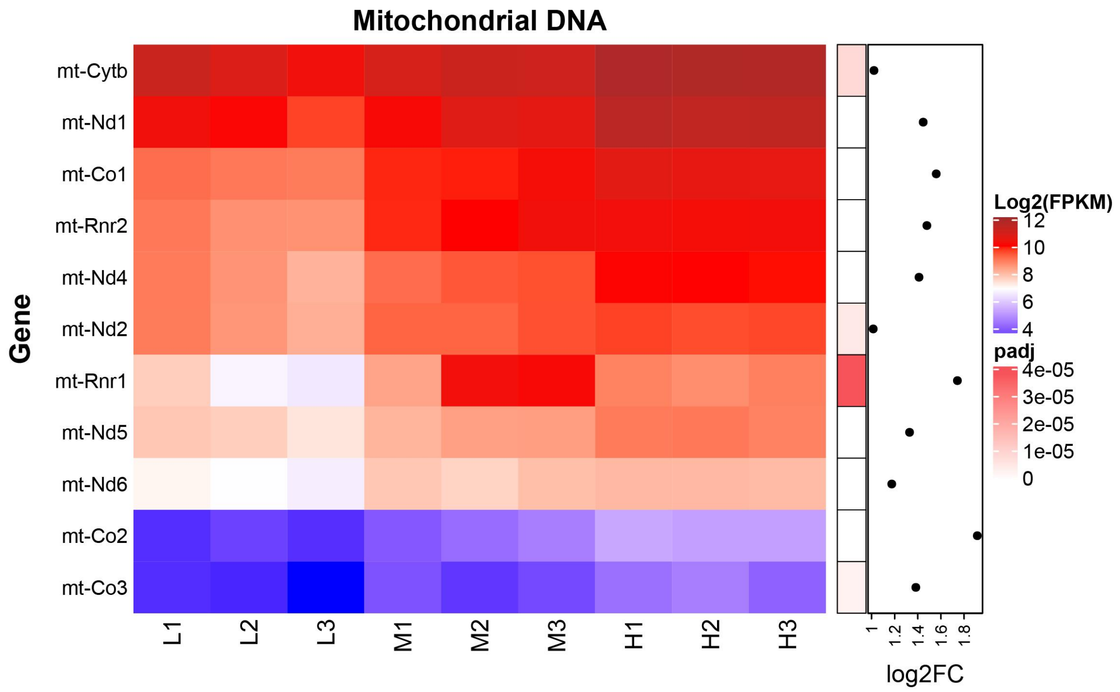

Supplementary Figure 4. The heatmap of mitochondrial DNA in profile 9 module. L, 2.88×108 v.g.; M, 2.88×109 v.g.; H, 2.88×1010 v.g..

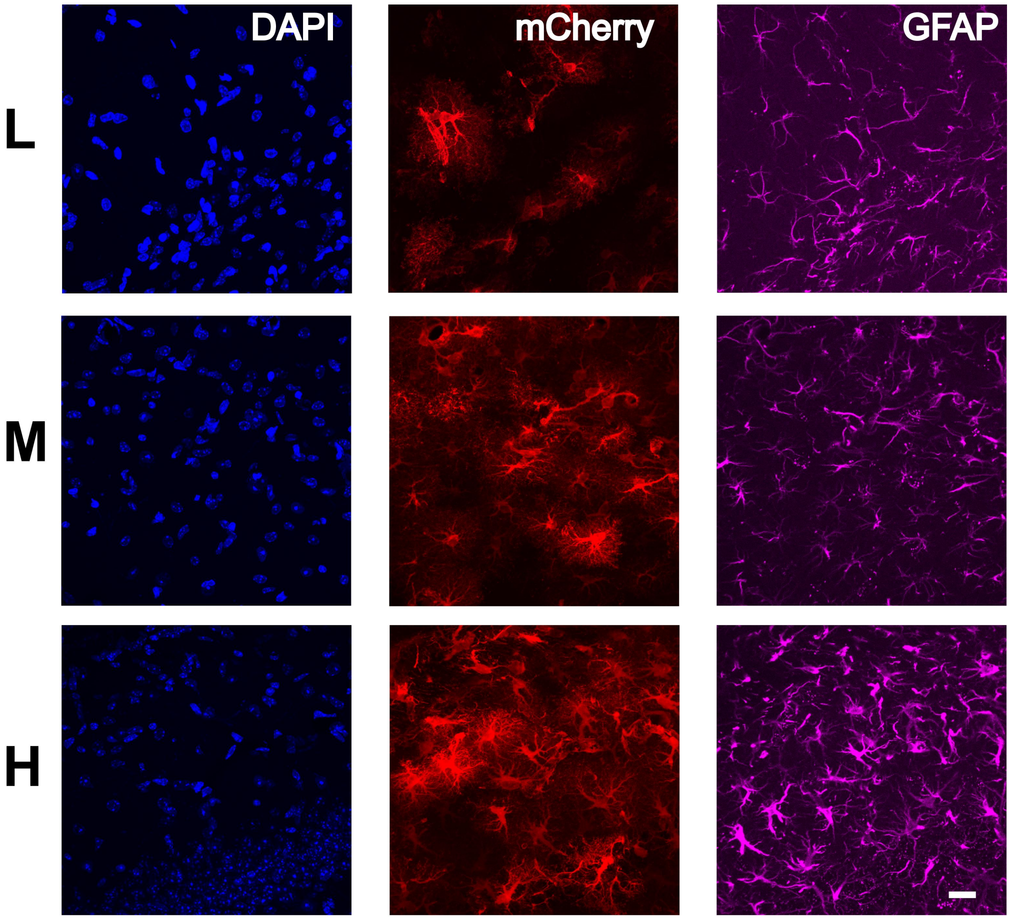

Supplementary Figure 5. High-dose rAAV5-mCherry induced reactive astrocytes in Aldh1l1-creER^{T2} mice. Scale bar, 20μm. L, 2.88 × 10^8 v.g.; M, 2.88 × 10^9 v.g.; H, 2.88 × 10^{10} v.g..
